# Transcriptomic landscape of CD8+ and CD4 + T-LGL leukemia revealed the distinct impact of *STAT3* and *STAT5B* activating mutations

**DOI:** 10.1038/s41375-025-02708-1

**Published:** 2025-07-28

**Authors:** Giulia Calabretto, Andrea Binatti, Antonella Teramo, Alessia Buratin, Gregorio Barilà, Vanessa Rebecca Gasparini, Cristina Vicenzetto, Enrico Gaffo, Elisa Rampazzo, Silvia Orsi, Elena Buson, Valentina Trimarco, Barbara Mariotti, Monica Facco, Flavia Bazzoni, Livio Trentin, Gianpietro Semenzato, Renato Zambello, Stefania Bortoluzzi

**Affiliations:** 1 Department of Medicine, Hematology and Clinical Immunology Unit, University of Padova, Padova, Italy; 2 Veneto Institute of Molecular Medicine (VIMM), Padova, Italy; 3 Department of Molecular Medicine, University of Padova, Padova, Italy; 4 Department of Biology, University of Padova, Padova, Italy; 5 Hematology Unit, San Bortolo Hospital, Vicenza, Italy; 6 Section of General Pathology, Department of Medicine, University of Verona, Verona, Italy; 7 Department of Surgery, Oncology and Gastroenterology, University of Padova, Padova, Italy

**Keywords:** Chronic lymphocytic leukaemia, Cytotoxic T cells

## Abstract

The biological basis of the high clinical heterogeneity of T-LGL Leukemia (T-LGLL) is not completely understood and effective therapies for this disease are lacking. Through RNA-Sequencing of purified T-LGLs we reveal gene expression profiles and pathway dysregulations in the major patient subgroups, defined by CD8+ or CD4+ phenotype and *STAT3*/*STAT5B* mutational status. Overall, T-LGLL patients exhibited a marked transcriptome dysregulation compared to controls. This was more pronounced in the most symptomatic CD8 + *STAT3*-mutated patients, which emerged as a distinct biological entity, separated from the other disease subgroups. Particularly, CD8 + *STAT3*-mutated cases displayed extensive down-regulation of genes, ultimately resulting in the de-repression of proliferation and cell cycle pathways. Among genes up-regulated in CD8 + *STAT3*-mutated cases we found *VCAM1*, the transcriptional repressor EZH2 and the p53-regulator MDM2 proto-oncogene, as well as the leukemogenesis-associated *PVT1* up-regulation, representing the first report of a long-non-coding RNA alterations in leukemic T-LGLs. The impact of *STAT5B* mutations on T-LGLs transcriptome was more limited and the overexpression of the *PIM1* serine/threonine kinase proto-oncogene was identified as one of the most relevant features of *STAT5B*-mutated CD4 + T-LGLL. This study significantly advances our understanding of T-LGLL pathogenesis, uncovering new oncogenic mechanisms within the distinct molecular subtypes of the disease.

## Introduction

T-cell large granular lymphocyte leukemia (T-LGLL) is a rare lymphoproliferative disease characterized by the clonal expansion of cytotoxic CD3 + T-Large granular lymphocytes (T-LGLs) [1]. The clinical and biological heterogeneity of T-LGLL is well recognized, ranging from chronic indolent proliferations to symptomatic and aggressive leukemias with poor outcome [2].

Nowadays, phenotypic [3] and genetic [4] features need to be considered in the classification of T-LGLL patients. According to the immunophenotype of the leukemic clone, two distinct subtypes of T-LGLL can be distinguished: the canonical CD8 + /CD4- T-LGL proliferation (CD8 + T-LGLL) and the less common CD4 + /CD8^dim/neg^ variant (CD4 + T-LGLL) [5], accounting for up to 30% of cases [6]. On a genetic basis, *STAT3* and *STAT5B* hyperactivating mutations represent the most frequent and clinically relevant lesions. Based on their unique association with the T-LGL clone’s immunophenotype [7] and clinical features, additional disease subgroups can be defined. *STAT3* mutations, predominantly detected in CD8 + T-LGLL patients [3, 6], are associated with the development of a symptomatic disease, mainly characterized by neutropenia and anemia [8, 9], leading to reduced overall survival of patients [6]. Conversely, *STAT5B* mutations, initially discovered in CD8 + T-LGLL with dismal outcome [10], are found at high frequency in CD4 + T-LGLL patients, which mostly present with indolent disease [6, 11, 12], similarly to *STATs*-mutation negative T-LGLL cases [6].

Several studies [13–15] have highlighted the crucial role of the JAK-STAT pathway in LGL survival network and its link with the disease clinical manifestations, addressing the molecular alterations that lead to its aberrant activation. Beside the occurrence of *STATs* mutations, cytokines [14, 16, 17], epigenetic [14, 16, 17] and microRNA dysregulations [18–20] have been reported. However, only a few studies have specifically evaluated the *STAT*-mediated gene expression changes in T-LGLL [10, 21–23], mostly through microarray profiling. More recently, additional insights have been provided into the transcriptomic features of *STAT3*-mutated T-LGLL [24], but a comprehensive gene expression profiling of the T-LGLL subtypes, distinguished by immunophenotype and *STAT3/STAT5B* genetic status, has never been performed. Most importantly, the oncogenic mechanisms occurring in each T-LGLL subtype, which likely account for the heterogeneous clinical courses of the disease, are still undefined.

In this study, through RNA-sequencing (RNA-seq) of highly purified T-LGLs from a sizable discovery cohort of T-LGLL patients, we identified gene expression profiles and activated/inhibited pathways with pathological relevance in different T-LGLL subtypes. These findings further clarify the molecular alterations that differentiate patients from healthy controls, as well as disease subgroups with distinct clinical outcomes.

## Material and methods

### Study design and ethical consent

RNA-seq analysis was performed in a discovery cohort of 20 T-LGLL patients and 5 healthy donors (HD) (Table 1). Additional 20 patients and 5 controls were used as an independent cohort for validations and functional experiments (Supplementary Table 1). Patients were recruited at the Hematology Unit of the Padua University Hospital and met the currently accepted diagnostic criteria for T-LGLL [25]. None of them had received treatment at the time of sample collection. The study was approved by the Ethic Committee for Clinical Trials of Padua (approval number: 4213/AO/17) and written informed consent was obtained from all the enrolled subjects, according to the Helsinki Declaration.

**Table 1:** Characteristics and molecular data of T-LGLL patients of the discovery cohort, equally divided in CD8  + (*N* = 10) and CD4  + (*N* = 10), with each group comprising half of cases with *STAT3* or *STAT5B* mutations, respectively.

CODE	Sex/Age (years)	T-LGLL	Immunophenotype of the T-LGL clone	TCR rearrangement	TCR Vb*	*STAT3*/*STAT5B* mutations
01	F/49	CD8 +	CD16 + /CD56-/CD57 +	C	17	*STAT3 Y640F*
02	F/67	CD8 +	CD16 + /CD56-/CD57 +	C	2	*STAT3 D661Y*
03	M/59	CD8 +	CD16 + /CD56-/CD57 +	C	ND*	*STAT3 Y640F*
04	F/84	CD8 +	CD16 + /CD56-/CD57 +	C	3	*STAT3 Y640F*
05	M/56	CD8 +	CD16 + /CD56-/CD57 +	C	2	*STAT3 D661V*
06	F/79	CD8 +	CD16-/CD56-/CD57 +	C	8	WT
07	M/79	CD8 +	CD16-/CD56 + /CD57 +	C	ND*	WT
08	F/81	CD8 +	CD16-/CD56 + /CD57 +	C	ND*	WT
09	M/63	CD8 +	CD16-/CD56-/CD57 +	C	1	WT
10	M/64	CD8 +	CD16-/CD56 + /CD57 +	C	13.1	WT
11	M/77	CD4 +	CD16-/CD56 + /CD57 +	C	5.1	*STAT5B N642H*
12	M/85	CD4 +	CD16-/CD56 + /CD57 +	C	13.1	*STAT5B Y665F*
13	F/77	CD4 +	CD16 + /CD56 + /CD57 +	C	ND*	*STAT5B T628S*
14	M/71	CD4 +	CD16-/CD56 + /CD57 +	C	13.1	*STAT5B Y665F*
15	M/75	CD4 +	CD16-/CD56 + /CD57 +	C	8	*STAT5B T628S*
16	F/64	CD4 +	CD16 + /CD56 + /CD57 +	C	3	WT
17	M/61	CD4 +	CD16-/CD56 + /CD57 +	C	13.1	WT
18	M/73	CD4 +	CD16-/CD56 + /CD57 +	C	ND*	WT
19	M/74	CD4 +	CD16-/CD56 + /CD57 +	C	13.1	WT
20	M/72	CD4 +	CD16-/CD56 + /CD57 +	C	1	WT

*C* clonal, *CD* Cluster of differentiation, *F* female, *M* male, *STAT3* Signal transducer and activator of transcription 3, *STAT5B* Signal transducer and activator of transcription 5B, TCR Vβ: T-cell Receptor Variable β-chain Region (TCR-Vβ) repertoire (*ND Not defined using the IOTest Beta Mark TCR-Vβ Repertoire kit, covering approximately 75% of Vb regions), WT: Wild type. TCR clonal rearrangement was evaluated by IdentiClone TCR γ gene Rearrangement Assay (Invivoscribe, San Diego, CA, USA).

### Immunophenotypic analysis

Immunophenotypic analysis was performed by flow cytometry on peripheral blood mononuclear cells (PBMC) of patients. Cells were stained with commercially available monoclonal antibodies (mAbs), as previously described [6]. The T-cell Receptor Variable β-chain Region (TCR-Vβ) repertoire of leukemic T-LGL was determined with the IOTest Beta Mark TCR-Vβ Repertoire kit (Beckman Coulter). Cells were analyzed using a FACS Canto II and data were processed with the BD FACSDiva and FlowJo softwares (BD).

### Mutational analyses

DNA was extracted from purified T-LGLs with the Gentra Puregene Cell Kit Plus (Qiagen). In the discovery and validation cohort, *STAT3* and *STAT5B* mutations were screened in the hot spot regions by Sanger sequencing, as previously reported [3]. Purified PCR products were sequenced using dye terminator technology and the ABI 3130 sequencer (Applied Biosystem). The most frequent variants were also investigated by Amplification Refractory Mutation System-polymerase chain reaction (ARMS-PCR), as previously described [14]. Patients from the discovery cohort were also screened for the presence of additional oncogenic variants reported to be associated with T-LGLL, by variant calling from RNA-seq data, as described in Supplementary Information.

### RNA-sequencing

Purified T-LGL were obtained from PBMC of patient and HD with magnetic micro-beads (Miltenyi Biotec) coated with monoclonal anti-human CD8, CD57 or CD56. Purity and viability of purified cells were assessed by flow cytometry. A threshold of at least 98% T-LGLs on all the acquired events coupled with contamination lower than 0.1% of monocytes was accepted.

Total RNA was extracted with the RNeasy Mini Kit (Qiagen) in combination with on-column DNase I digestion (RNase-Free DNase set, Qiagen). RNA quality was assessed using the RNA 6000 Nano and RNA 6000 PICO assays on a 2100 Bioanalyzer (Agilent). Sequencing libraries were prepared using a TruSeq Stranded Total RNA Kit adapted for long fragments (±550 bp) with the Ribo-Zero Gold rRNA Removal Kit (Illumina). Prepared libraries were run on a HiSeq3000 high-throughput sequencing system (Illumina) and paired-end reads were generated (315 million reads per sample, on average).

### Bioinformatics analysis

Assessment of RNA-seq data quality was performed for each sample with FASTQC software. RNA-seq data were analyzed for reads alignment and transcripts quantification by CircComPara2 pipeline [26], which quantifies gene expression using StringTie. The estimates were summarized with the tximport package.

Genes with less than 10 reads in at least 5 samples were considered not expressed and removed from the analysis. Expression values were normalized for removing batch effects and other unwanted variations in the sequencing by a Surrogate Variable Analysis (sva package). Differential expression of linear transcripts was assessed using DESeq2 and set an adjusted *p*-value < = 0.01 as the significance threshold for all contrasts. The variation coefficient (VC) was used to rank genes by expression profile variability.

Gene clustering analysis was obtained by the degPatterns function of the DEGreport R package. This analysis was applied to the normalized counts data of differentially expressed genes. The divisive hierarchical clustering was conducted using the *diana* function in R, based on the Kendall rank pairwise correlation coefficient between genes. Only clusters with at least 5 genes were retained.

Gene set enrichment analysis (GSEA) was performed using the R packages “clusterProfiler” [27], “enrichplot” and “ViSEAGO” [28].

### Transcript and protein expression quantification

Methods for quantification of selected gene transcripts, by RT-qPCR, are detailed in Supplementary Information.

### Treatments and evaluation of cell viability

PBMC from patients (2 × 10^6^ cells/mL) were cultured in RPMI-1640 medium (EuroClone), supplemented with 10% FCS (Sigma-Aldrich), 2 mM Glutamine, 25 mM Hepes, 100 U/mL penicillin and 100 mg/mL streptomycin (EuroClone) and grown in 5% CO_2_ at 37 °C. Cells were treated with Stattic 2.5 μM, a selective STAT3 inhibitor. T-LGLs viability was evaluated by Annexin V (BD Pharmingen) staining and flow cytometry.

## Results

### Key aberrant expression patterns differentiate the T-LGLL subgroups

**Fig. 1:**
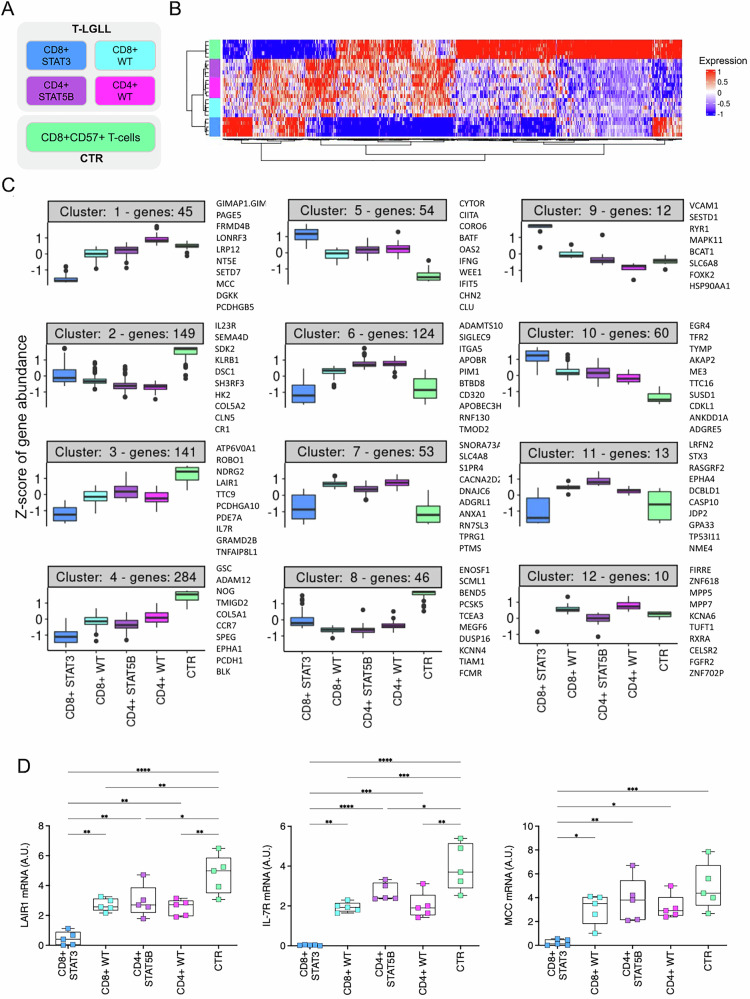
Study design and sample clustering analysis based on gene expression profiles. **A** Study design and T-LGLL patient sample groups. **B** Heatmap (standardized expression, distance etc) and (**C**) Clustering of the expression profiles of the 1000 most variable genes among those significantly differentially expressed when comparing each of the four T-LGLL groups with respect to CTR samples (DESeq2 *p*-value ≤ 0.01). In (**C**) the y-axis shows the Z-score, thus values are centered to the mean and scaled to the standard deviation by each gene. **D** Boxplot of the relative gene expression quantified by RT-qPCR (Delta Delta Ct (DDCt) method; GAPDH used as reference gene; Mean ± SD shown; A.U., Arbitrary Units) in a validation group of patients (5 samples/group for each gene), characterized for immunophenotype and STAT3/STAT5B mutational status, and CTR samples. *p < 0.05; **p < 0.01; ***, p < 0.001.

High-depth RNA-seq data were obtained from a discovery cohort of 20 T-LGLL patients (GEO GSE228868), equally stratified into four distinct T-LGLL subtypes (5 patients/group), defined based on the immunophenotype of the leukemic clone (CD8+ or CD4 + ) and *STAT3*/*STAT5B* mutational status (Table 1, Fig. 1A). Cytotoxic CD8 + CD57 + T-cells from 5 healthy controls (CTR) (Fig. 1A) were included as a normal counterpart, being the closest cell of origin of all the four T-LGLL subtypes considered in the study (Supplementary Fig. 1). Considering the clinical features of the enrolled patients (Table 2), all CD8 + *STAT3*-mutated cases were neutropenic, in line with previously reported series [3, 6]. Variant calling from RNA-seq data revealed a subclonal *STAT5B* variant in one *STAT5B*-mutated case, as well as oncogenic variants in seven additional genes across different patients, all previously reported in T-LGLL, obtaining a better characterization of the patient genetic profile (Supplementary Methods and Supplementary Table 2).

**Table 2:** Clinical features of the 20 T-LGLL patients of the discovery cohort.

T-LGLL subgroup	CODE	WBC 10^9^/L	ALC 10^9^/L	T-LGL 10^9^/L	ANC 10^9^/L	Hb g/L	PLT 10^9^/L
**CD8** + **T-LGLL** ***STAT3*-mutated**	01	5.44	4.31	2.80	0.34	133	193
	02	12.99	11.87	8.07	0.64	127	162
	03	4.42	2.80	1.68	0.95	142	266
	04	5.56	4.23	2.07	0.65	129	233
	05	5.69	3.90	2.22	0.88	140	141
**CD8** + **T-LGLL** **WT**	06	6.80	3.59	1.80	2.92	130	151
	07	8.81	4.25	2.80	3.37	152	208
	08	9.19	6.80	5.10	1.84	130	186
	09	6.87	3.50	1.40	2.58	155	311
	10	7.01	1.80	1.19	4.48	131	223
**CD4** + **T-LGLL** ***STAT5B*-mutated**	11	12.61	6.36	3.18	4.27	148	266
	12	9.53	3.60	2.41	3.42	132	164
	13	5.59	3.43	2.16	1.92	134	190
	14	14.60	5.05	2.78	2.52	146	217
	15	19.06	12.15	11.18	4.09	162	199
**CD4** + **T-LGLL** **WT**	16	14.03	4.27	2.18	6.63	148	330
	17	7.35	4.94	2.47	1.70	150	159
	18	9.84	5.85	4.91	3.72	148	263
	19	15.48	10.98	8.56	3.92	150	164
	20	16.01	3.91	1.92	3.07	143	234

*ALC* Absolute lymphocyte count, *ANC* absolute neutrophil count, *Hb* Hemoglobin, *PLT* Platelets, *T-LGL* T-Large Granular Lymphocytes, *WBC* white blood count.

The transcriptomic profiles of T-LGLL and CTR samples (29,947 genes considered) were then investigated to look for both disease-specific dysregulation and transcriptional differences across patient subgroups. A first comparison of the gene expression profiles of the four T-LGLL groups in relation to CTR (Fig. 1A, B), using stringent criteria, led to the identification of 2335 differentially expressed genes (DEGs, FDR ≤ 0.01; Supplementary Table 3). To capture the main patterns of expression, unsupervised sample clustering was performed on the most variable 1000 DEGs, resulting in the definition of twelve clusters, each containing at least 5 genes (Fig. 1B, C). In addition to a clear separation of patients from CTR, sample clustering also separated CD8 + *STAT3*-mutated from all the other T-LGLL cases, pointing toward a more marked dysregulation of the transcriptome in link with hyperactivating *STAT3* mutations.

Two of the largest gene clusters (3 and 4, including 141 and 284 genes, respectively) were down-regulated in T-LGLL with respect to CTR, particularly in the CD8 + STAT3 group (f.i. *CCR7* was down-regulated in all patients, whereas *LAIR1* and *IL7R* particularly in CD8 + *STAT3*-mutated cases). Clusters 1 (45 genes, f.i. *MCC, GIMAP1.GIMAP5* readthrough, *PAGE5*, *LRP12*) and 12 (10 genes, f.i *FIRRE*, *ZNF618*) were down-regulated specifically in the presence of *STAT3* mutation. Several genes were similarly down-regulated in all T-LGLL, somewhat less markedly in *STAT3*-mutated cases, e.g. cluster 2 (149 genes, including key receptors like *IL23R* and *KLRB1*) and cluster 8 (46 genes, including tumor suppressors like *BEND5* and *TCEA3*). On the other hand, genes up-regulated in T-LGLL, particularly in the presence of *STAT3* mutations, were grouped in clusters 5 (54 genes, including the previously reported *ZBTB46* [24] and *CIITA*, *BATF*, *IFNG*), 9 (12 genes, f.i. *VCAM1*, *RYR1*) and 10 (60 genes, f.i. *EGR4*, *ME3*). Of note, genes of clusters 6, 7 and 11 (124, 53 and 13 genes, respectively) showed up-regulation in most LGLL samples, excluding the most symptomatic *STAT3*-mutated cases.

The down-regulation of MCC (cluster 1), LAIR1 and IL7R (cluster 3) in T-LGLL and their even more marked reduction in *STAT3*-mutated cases was confirmed by RT-qPCR (Fig. 1D) in an independent cohort of patients (Supplementary Table 1), supporting the robustness of our data.

### *STAT3*-mutated CD8 + T-LGLL are characterized by a unique transcriptomic signature

**Fig. 2:**
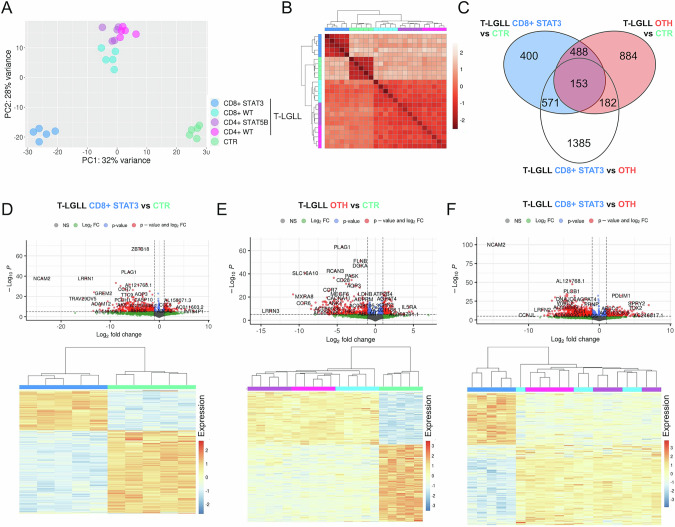
Gene expression dysregulation in T-LGLL defines two main subgroups of cases. **A** PCA and **B** hierarchical clustering of samples according to gene expression profiles correlation-based distance. **C** Overlap of the genes differentially expressed when comparing STAT3 CD8 + LGLL with CTR samples, OTH LGLL with CTR samples and STAT3 CD8 + LGLL with OTH. **D**–**F** Volcano plots and heatmaps of significantly differentially expressed genes in each comparison.

Unsupervised principal components analysis and clustering of the whole gene expression profiles clearly separated T-LGLL from CTR samples (Fig. 2A, B). The CD8 + STAT3 T-LGLL group, characterized by a more symptomatic disease, was distinguished from the other three subtypes (from now on considered as a single group, named “others”, OTH) and from CTR, confirming a peculiar profile of *STAT3*-mutated cases, in line with the results reported in Fig. 1B–D. Considering these data and the clinical features of CD8 + *STAT3*-mutated cases, additional studies were focused on the pairwise comparisons of two main entities, i.e., CD8 + STAT3 and OTH T-LGLL, with respect to CTR and to each other (Fig. 2C; Supplementary Table 4).

CD8 + STAT3 T-LGLL had a significantly altered transcriptome, witnessed by a total of 1612 detected DEGs, with the majority (69%) being down-regulated in leukemic T-LGL compared to CTR (Fig. 2C, D). A similar number of genes (1707) were dysregulated (60% down-regulated) in OTH T-LGLL (Fig. 2E). Direct comparison of CD8 + STAT3 with OTH T-LGLL enabled the identification of 2291 DEGs, mostly (61.2%) down-regulated in the former (Fig. 2F). As shown in the Venn diagram (Fig. 2C), 641 genes were aberrantly expressed in both patient groups, with 488 being similarly altered and 153 showing differences between CD8 + STAT3 and OTH T-LGLL samples. Of note, most of the commonly dysregulated genes were concordantly up- or down-regulated in all cases. However, a more marked dysregulation in the presence of *STAT3* genetic lesions was observed for most of the genes with aberrant expression (Supplementary Fig. 2).

**Fig. 3:**
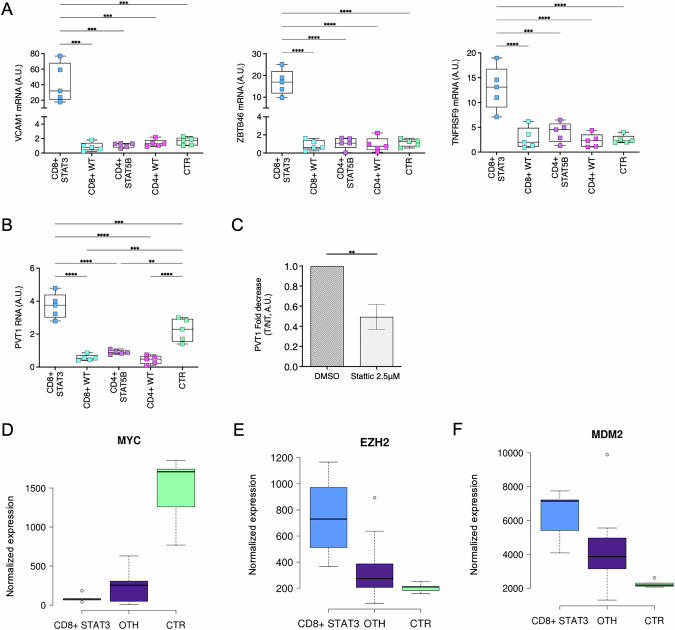
PVT1 upregulation in *STAT3*-mutated T-LGLL. **A** Boxplot of the relative gene (VCAM1, ZBTB46, TNFRSF9) expression quantified by RT-qPCR (Delta Delta Ct (DDCt) method; GAPDH used as reference gene; Mean ± SD shown; A.U. Arbitrary Units) in a validation group of patients (5 samples/group for each gene, characterized for immunophenotype and STAT3/STAT5B mutational status) and CTR samples. *p < 0.05; **p < 0.01; ***p < 0.001. **B** Boxplot of PVT1 expression according to RT-qPCR analysis; Mean ± SD are shown). **C** PVT1 expression, analyzed by RT-qPCR, in CD8 + T-LGLs from STAT3 mutated patients cultured for 6 h with 5 μM Stattic or DMSO as control (*N* = 4; expression is reported as fold decrease relative to DMSO-treated cells, after GAPDH normalization. Mean ± SD are shown). **D** Boxplot of MYC expression according to RNA-seq quantification. **E** Boxplot of EZH2 expression according to RNA-seq quantification. **F** Boxplot of MYC expression according to RNA-seq quantification.

Distinct genes were specifically up-regulated in *STAT3*-mutated CD8 + T-LGLL as compared to CTR. The most dysregulated ones included STAT3 transcriptional targets, such as VCAM1, and other genes like ZBTB46 [24] and TNFRSF9, whose differential expression was confirmed by RT-qPCR in the extended cohort (Fig. 3A). Among the class of dysregulated long non-coding RNAs (lnc-RNAs), it is noteworthy to mention the Plasmacytoma variant translocation 1 (PVT1), due to its known association with oncogenic mechanisms in solid cancers and other hematologic malignancies [29]. Firstly, we confirmed the specific up-regulation of PVT1 observed in *STAT3*-mutated cases in an independent cohort of T-LGLL cases (Fig. 3B). In addition, by treating samples (PBMC) from *STAT3*-mutated T-LGLL patients with Stattic, a STAT3 inhibitor, we observed a significant reduction of PVT1 RNA (Fig. 3C), consistent with the lower PVT1 expression observed in T-LGLL cases with STAT3 wild-type. This finding suggests a dependency of PVT1 expression on STAT3 hyperactivation.

Next, we explored some of the putative mechanisms by which PVT1 might contribute to the disease pathogenesis. We excluded a PVT1-mediated overexpression of MYC [30], being the expression of this oncogene significantly down-regulated in *STAT3*-mutated T-LGLL as compared to CTR (Fig. 3D). Thus, we considered a possible interaction between PVT1 and the Enhancer Of Zeste 2 Polycomb Repressive Complex 2 Subunit (EZH2) [31], which plays a role in epigenetic remodeling, leading to transcriptional repression of target genes. This hypothesis, based on the massive gene downregulation observed in *STAT3*-mutated patients, is supported by the significant upregulation of EZH2 observed in malignant cells compared to CTR samples (Fig. 3E). In addition, we found a significantly increased expression of the murine double minute 2 (MDM2), a negative regulator of the tumor suppressor p53, in CD8 + STAT3 cases with respect to OTH T-LGLL and CTR (Fig. 3F). This finding might also support a role of PVT1 in suppressing cell apoptosis and enhancing cell proliferation through the stabilization of MDM2 mRNA [31].

### Activation of pro-survival pathways is the hallmark of *STAT3-*mutated T-LGLL whereas GPCR signaling suppression characterizes *STAT3* mutation negative cases

**Fig. 4:**
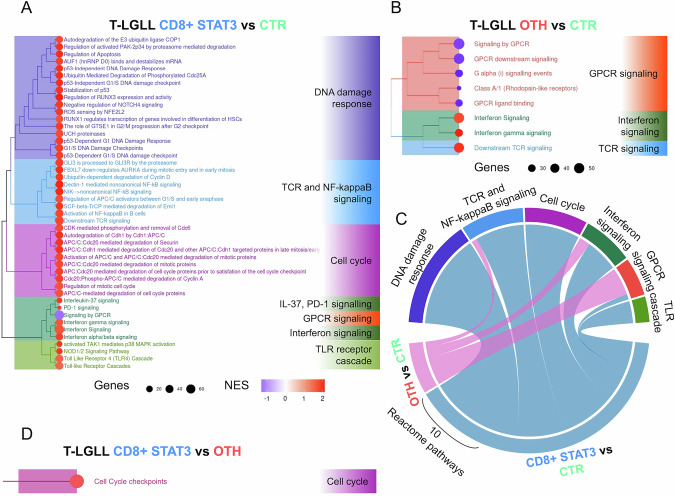
Pathway dysregulation in T-LGLL subgroups. Clustering of the Reactome pathways significantly enriched according to GSEA comparing (**A**) STAT3 CD8 + LGLL with CTR samples. **B** OTH LGLL with CTR samples and (**C**) Circos plot showing the overlap of pathways significantly enriched in the group comparisons above (the width of the belts connecting comparisons to pathways is proportional to the number of pathways enriched). **D** Clustering of the Reactome pathways significantly enriched according to GSEA comparing STAT3 CD8 + LGLL with OTH. In (**A**–**D**) positive and negative values of the normalized enrichment score (NES) indicate the extent of pathway overrepresentation among up- and down-regulated genes, respectively, and the dot size in proportion to the number of genes involved.

Understanding the pathogenetic relevance of the extensive gene dysregulation observed in T-LGLL required further investigation of the biological functions and signaling pathways altered in the disease. In this regard, gene set enrichment analysis (GSEA) highlighted that distinct signaling pathways are activated in the CD8 + STAT3 and OTH T-LGLL compared with CTR, also revealing the differences between the two main patient subgroups in terms of pathway activation. CD8 + STAT3 T-LGLL presented alterations in 46 Reactome pathways, which were clustered in 5 main groups (DNA damage response; TCR and NF-kB signaling; Cell cycle; Interferon, IL-37, PD-1 and G protein-coupled receptor signaling (GPCR) signaling; TLR receptor cascade) (Fig. 4A). All these pathways were activated in *STAT3-*mutated samples, except for GPCR signaling. Differently, OTH T-LGLL showed only eight aberrantly active Reactome pathways, including 3 activated pathways primarily related to Interferon and TCR signaling, and 5 inhibited pathways, all belonging to the GPCR signaling (Fig. 4B).

The extensive activation of pro-survival pathways observed in CD8 + STAT3 T-LGLL was primarily due to the enrichment of genes involved in DNA-damage response (both p53-dependent and -independent checkpoint regulation) and in cell cycle regulation, particularly mitotic cycle modulation via APC/C. Specifically, we found 17 and 10 significantly enriched pathways with positive NES, respectively. Noteworthy, the regulation of apoptosis (with the overexpression of anti-apoptotic genes, such as ARHGAP10 and RhoA) and protein degradation functions (e.g., ubiquitin and several proteasome subunits) were among the most hyperactive pathways in *STAT3*-mutated cases.

Overall, the TCR and Interferon signaling activation were shared by all T-LGLL cases, although extensively in the presence of *STAT3* activating mutations (Fig. 4C). Suppression of GPCR was a common trait of patients, nevertheless more inhibited in OTH T-LGLL cases, that were characterized by a defective expression of genes such as CCR2, CCR7, CCR9, WNT11, TIAM genes. Interestingly, and in line with above-described observations, only one enriched pathway emerged in the direct comparison of CD8 + STAT3 and OTH T-LGLL, i.e. the activation of the cell cycle checkpoint pathway represents a distinctive feature of *STAT3*-mutated T-LGLL (Fig. 4D).

### PIM1 over-expression is a key feature of *STAT5B-*mutated CD4 + T-LGLL

**Fig. 5:**
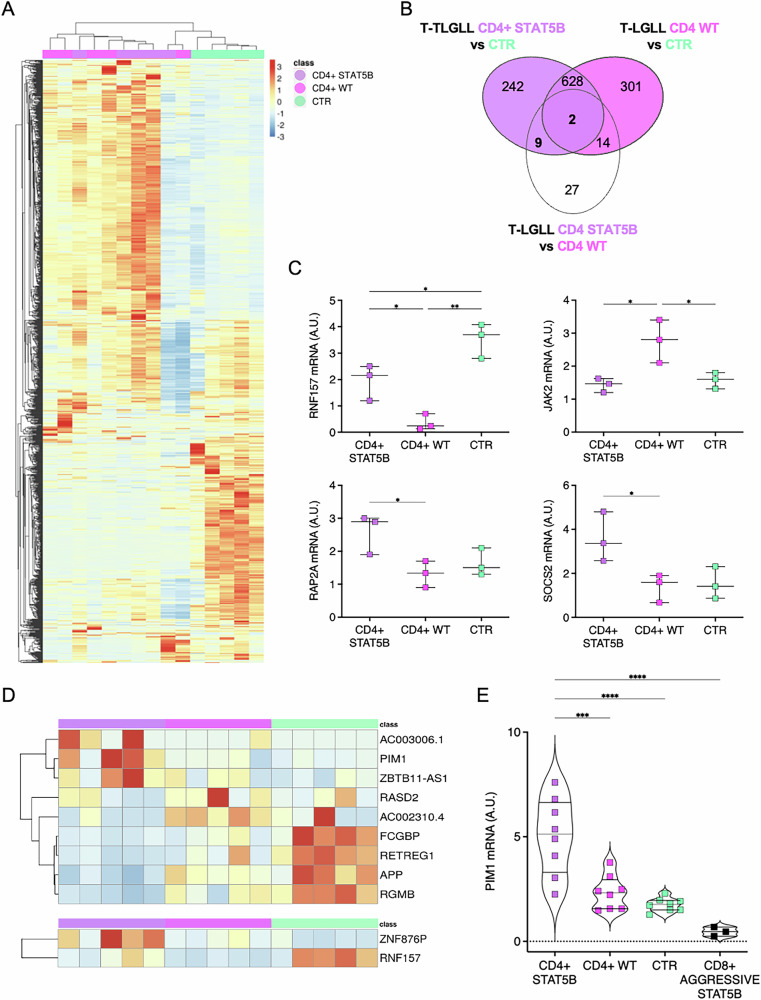
CD4 + T-LGLL transcriptomic alterations features. **A** Heatmap of the expression profiles and (**B**) overlap of the genes significantly differentially expressed (DESeq2 *p*-value < = 0.01) when comparing STAT5b mutated and not mutated CD4 T-LGLL among themselves and across the normal counterpart. **C** Boxplot of the relative gene (RNF157, JAK2, RAP2A, SOCS2) expression quantified by RT-qPCR (Delta Delta Ct (DDCt) method; GAPDH used as reference gene; Mean ± SD shown; A.U. Arbitrary Units) in a validation group of patients (3 samples/group for each gene, characterized for immunophenotype and STAT3/STAT5B mutational status) and CTR samples. *p < 0.05; **p < 0.01. **D** Closeup of the expression profiles of genes altered more specifically in link with STAT5b lesions (bold numbers in panel **B**). **E** Boxplot of the relative PIM1 expression quantified by RT-qPCR (Delta Delta Ct (DDCt) method; GAPDH used as reference gene; Mean ± SD shown; A.U. Arbitrary Units. *p < 0.05, ***p < 0.001, ***p < 0.0001.

Next, we explored CD4 + T-LGLL patients, further distinguished in two subgroups based on the presence of *STAT5B* mutations. Differential expression tests of pairwise comparisons between CD4 + *STAT5B*-mutated, CD4 + WT and CTR were performed (Fig. 5A), identifying a total of 881 and 945 dysregulated genes, respectively (Fig. 5A, B). A slight tendency towards a prevailing gene down-regulation was observed, with 56% of genes less expressed in T-LGLL cases in both comparisons. Most (630) DEGs were commonly dysregulated in both groups, irrespective of the presence of *STAT5B* lesions. Nevertheless, differences between *STAT5B*-mutated and WT subgroups could be observed (Fig. 5A, B). In line, the direct comparison of CD4 + STAT5B versus CD4 + WT T-LGLL cases identified 52 DEGs, with 27 not significantly altered in comparison with the normal counterpart (e.g. the experimentally validated *RAP2A*, *SOCS2*, Fig. 5C) and 25 dysregulated in at least one group. In detail, 2 genes were dysregulated in both comparisons: *ZNF876P* was up-regulated in CD4 + T-LGLL, but more in *STAT5B*-mutated cases, whereas *RNF157* (Fig. 5C) was down-regulated, particularly in WT cases. Fourteen genes were aberrantly expressed only in CD4 + WT with respect to CTR, including *JAK2* (Fig. 5C). Nine differentially expressed genes were found in the comparison of CD4 + *STAT5B*-mutated and CTR. Among the features linked to the *STAT5B* mutation (Fig. 5D), the up-regulation of *PIM1*, confirmed in the extended cohort (Fig. 5E), particularly attracted our interest. Taking advantage of three available T-LGLL samples diagnosed with the rare and highly aggressive CD8 + *STAT5B*-mutated T-LGLL form (Supplementary Table 5), we investigated PIM1 expression also in this additional T-LGLL subgroup. Our goal was to evaluate whether PIM1 could represent a molecular alteration linked to the occurrence of *STAT5B* mutations. Notably, our data revealed the PIM1 up-regulation represents an alteration uniquely present in the CD4 + STAT5B-mutated T-LGLL, characterized by an indolent clinical course. In contrast, a defective expression of PIM1 was observed in T-LGLs from aggressive CD8 + *STAT5B*-mutated patients, compared to both CD4 + T-LGLL and CTR (Fig. 5E).

## Discussion

In this study we present novel insights into the transcriptomic features of T-LGLL, highlighting the distinction between major T-LGLL subsets based on molecular data, particularly *STAT3* and *STAT5B* mutations, which correlate with the different clinical courses observed in patients.

Through RNA-seq profiling of purified T-LGLs, we extend previous NGS-based findings in T-LGLL [24], providing new data on gene expression patterns and pathway activations in both CD8+ and CD4 + T-LGLL subtypes, further stratified by the genetic status of *STAT3* and *STAT5B*. By including distinct immunophenotypic and molecular disease subtypes, we found that *STAT3*-mutated patients, which are typically the most symptomatic and treatment-requiring [32], exhibit distinct transcriptome profiles compared to all other T-LGLL subgroups considered in this study. This finding underscored the need to further investigate gene expression patterns and dysregulated pathways within these two main T-LGLL entities. Interestingly, they presented only a partial overlap of genes with aberrant expression, with the majority of commonly dysregulated transcripts showing more pronounced alterations in presence of *STAT3* genetic lesions.

The interpretation of the pathogenetic relevance of the extensive gene dysregulation observed in T-LGLL, particularly in *STAT3*-mutated cases, required further investigation of signaling pathways alteration. The predominant activation of DNA damage response and cell cycle pathways might result from the widespread downregulation of genes that potentially inhibit these pathways. For instance, we observed a defective expression of LAIR1 (Leukocyte Associated Immunoglobulin Like Receptor 1), which exerts a constitutive negative regulatory role of immune cell cytolytic functions. Additionally, LAIR1 plays a role in the recruitment and activation of SHP-1 [33], a tyrosine phosphatase that functions as a negative regulator of several pathways, including the JAK/STAT3 axis [34]. Among genes with defective expression, it is also noteworthy to mention MCC (Mutated In Colorectal Cancers), which is specifically down-regulated in *STAT3*-mutated cases. Functional studies in colorectal cancer revealed that MCC is a candidate tumor suppressor that negatively regulates cell cycle progression, cell proliferation and migration, and is required for DNA damage response [35]. Loss-of-function mutations or decreased expression of this gene has also been reported in many other solid tumors [36].

Our observation of a global reduction of gene expression could be consistent with epigenetic disturbances in T-LGLL cells, which are described as a general hallmark of the disease. Indeed, several down-regulated genes in our data-set were found to have hypermethylated promoter and regulatory regions [37]. For instance, the observed defective expression of the experimentally validated *IL7R* and of *BCL11B* is in line with previous findings [37], corroborating the robustness of our data. Similarly, the observed up-regulation of other genes (e.g. IL7) could be explained by the respective gene promoter hypomethylation [37].

Otherwise, the extensive gene expression dysregulation observed in *STAT3*-mutated cases can also be due to the direct STAT3 transcriptional activity. This is exemplified by one of the most up-regulated transcripts, i.e., *VCAM1*, a STAT3-target involved in leukocyte-endothelial cell adhesion and signal transduction, which may also play a role in the development of rheumatoid arthritis [38], an autoimmune disorder often reported in association with T-LGLL.

Overall, the observed impact of *STAT3* mutations on transcriptomic alterations in the analysed patients, predominantly characterized by clonal *STAT3* mutations, aligns with previously published single-cell RNA-seq data, which demonstrate that *STAT3-*mutated clonotypes exhibit deregulated expression of genes involved in T cell survival and cytokine signaling compared to their unmutated counterparts within the same patient [39].

Besides identifying differentially expressed protein-coding genes, a novel aspect of this study is the first-time investigation of long non-coding RNAs in LGLL patients. In particular, our attention was attracted by the aberrantly expressed PVT1, which plays oncogenic roles (mainly due to increased copy number and overexpression) in several types of solid and hematological cancers [30]. Our results showed that PVT1 overexpression is specific for *STAT3*-mutated T-LGLL, also providing evidence of a STAT3-dependency, similarly to what observed in colorectal cancer, where a STAT3-PVT1 feed-forward regulatory loop was described [40]. Most importantly, we speculate a possible role of PVT1 overexpression in sustaining malignant cell features in the most symptomatic group of cases. Functional studies on PVT1 are complicated by its multiple transcript variants, both linear and circular. Herein, we addressed some putative mechanisms by which the lnc-PVT1 might contribute to T-LGLL pathogenesis. Due to their close proximity at the 8q24 locus, PVT1 and the oncogene *MYC* are often considered twin players and a positive interaction feedback loop has been demonstrated in other hematological malignancies [29]. However, our finding of MYC down-regulation in leukemic T-LGLs ruled out this hypothesis. Further analyses were aimed to evaluate PVT1 putative interaction with EZH2, playing a role in gene silencing. The finding of an upregulation of EZH2 support a possible involvement of the PVT1/EZH2 complex in the epigenetic remodeling occurring in T-LGLL. In addition, PVT1 might also promote the stabilization of other target genes, including MDM2, that we found upregulated in CD8 + STAT3 T-LGLL. MDM2 plays a critical role in p53 regulation, and its aberrant expression may result in the suppression of cell apoptosis and enhanced cell proliferation, as emerged from the GSEA.

Another highlight of this study was the identification of PIM1 overexpression in *STAT5*-mutated CD4 + T-LGLL cases. CD4 + T-LGLL represents a still less characterized subset of disease, being a rare and clinically indolent variant. In the genetic landscape of the disease, *STAT5B* mutations are of particular interest due to their opposite clinical significance they can assume. Indeed, in the CD8 + T-LGLL subtype they are associated with an aggressive and chemo-resistant disease with a poor prognosis. Our RNA-seq data revealed a limited biological impact of *STAT5B* mutations, as they do not significantly modify the transcriptional profile within the patient cohort. This suggests that the molecular impact of *STAT5B* mutations may be balanced by compensatory molecular mechanisms. In this regard, one of the most interesting findings is the peculiar over-expression of PIM1 observed in the group of mutated patients, which may play a central role in regulating STAT5B axis. Previous evidence has demonstrated PIM1 interaction with members of the SOCS family, contributing to their inhibitory activity on STAT5 [41]. In the context of T-LGLL, we can speculate that PIM1 represents a crucial crossroad in the negative feedback regulation of STAT5B and that a supraphysiological level of PIM1 could mitigate the effect of STAT5B hyperactivating mutations, resulting in a clinically indolent disease as observed in CD4 + T-LGLL. Our finding of a defective PIM1 expression in a small cohort of aggressive CD8 + T-LGLL supported the reasonableness of our hypothesis, along with the finding of PIM1 down-regulation in another aggressive mature T-cell leukemia, i.e. T-cell prolymphocytic leukemia [25]. Further analyses are required to confirm the data in a larger cohort of cases and to evaluate whether the defective expression of PIM1 might become a novel potential therapeutic target.

Lacking robust biological rationale for personalized therapy, T-LGLL remains an incurable disease, leaving patients with limited, nonspecific therapeutic options. A more comprehensive understanding of the underlying biological mechanisms driving the different clinical courses and outcomes of patients is crucial for advancing the development of novel therapeutic approaches.

Our results have made significant contributions to the knowledge of transcriptomic abnormalities in leukemic LGL, particularly regarding the major clinically relevant disease subtypes. We envisage that future research will delve deeper into the T-LGLL transcriptome, possibly focusing on diverse classes of non-coding RNAs that are promising sources of potential therapeutic targets and in turn on the design of innovative RNA-based treatment strategies for T-LGLL patients.

## Supplementary information

Supplementary information_Rev1

Suppl_table_1_rev

Suppl_table_2_rev

Suppl_table_3_rev

Suppl_table_4_rev

Suppl_table_5_rev

## Data Availability

RNA-sequencing data are available at GEO under accession number GSE228868. Databank URL: https://www.ncbi.nlm.nih.gov/geo/.
