# Supplementary material for: Transcriptomic landscape of CD8+ and CD4 + T-LGL leukemia revealed the distinct impact of STAT3 and STAT5B activating mutations
Source: Leukemia. 2025 Jul 28;39(10):2364–74. doi: 10.1038/s41375-025-02708-1 (PMC12463674; doi:10.1038/s41375-025-02708-1)
Supplement: Supplementary file 1 — Supplementary information_Rev1 [file 41375_2025_2708_MOESM1_ESM.docx]

**Transcriptomic landscape of CD8+ and CD4+ T-LGLL revealed the distinct impact of *STAT3* and *STAT5B* activating mutations**

Giulia Calabretto^1,2*^, Andrea Binatti^3*^, Antonella Teramo^1,2^, Alessia Buratin^3,4^, Gregorio Barilà^5^, Vanessa Rebecca Gasparini^1,2^, Cristina Vicenzetto^2^, Enrico Gaffo^3^, Elisa Rampazzo^1,2^, Silvia Orsi^1,4^, Elena Buson^1,2^, Valentina Trimarco^1^, Barbara Mariotti^6^, Monica Facco^1,2^, Flavia Bazzoni^6^, Livio Trentin^1^, Gianpietro Semenzato^1,2^, Renato Zambello^1,2$^ and Stefania Bortoluzzi^7$^

^1^Department of Medicine, Hematology and Clinical Immunology Unit, University of Padova, Padova, Italy

^2^ Veneto Institute of Molecular Medicine (VIMM), Padova, Italy

^3^Department of Molecular Medicine, University of Padova, Padova, Italy

^4^ Department of Biology, University of Padova, Padova, Italy

^5^ Hematology Unit, San Bortolo Hospital, Vicenza, Italy

^6^ Section of General Pathology, Department of Medicine, University of Verona, Verona, Italy

^7^ Department of Surgery, Oncology and Gastroenterology, University of Padova, Padova, Italy

* Co-first authors

$ Corresponding & Co-last author

**Supplementary information**

**Variant Calling from RNA-seq data**

To identify variants from RNA-seq data, sequencing reads were aligned to the human reference genome (hg38) using STAR (v2.7.5) in Two-Pass mode. Alignment processing was carried out following the Genome Analysis Toolkit (GATK v4.2.4) best practices for RNA-seq variant calling. Single nucleotide variant (SNV) detection was performed using Mutect2[^1^](https://paperpile.com/c/aLk9ty/Izfs) (GATK v4.2.4), while insertion/deletion (InDel) detection was conducted with RNAindel[^2^](https://paperpile.com/c/aLk9ty/4kvU) (v3.3.5). Variant annotation was performed using SnpEff and SnpSift with reference data from dbSNP (build 155), ClinVar, COSMIC (v95), GnomAD (v4.0), and REDIportal (hg38) and Cancer Genome Interpreter (CGI). Variants overlapping known RNA editing sites or splice junctions (within 5 nucleotides, as defined by RegTools) were excluded. Variants with a population allele frequency below 1%, a variant allele frequency (VAF) of at least 5% in the tumor sample and in sites covered by at least 20 reads were considered. We focused our analysis on a custom list of genes known to be mutated in T-LGLL according to literature^3-6^ and reported only those variants with a predicted functional impact HIGH or MODERATE and classified as "oncogenic" by CGI analysis.

**Transcript and protein expression quantification for validations**

Quantification of selected gene transcripts was assessed by RT-qPCR. Complementary DNA was generated from 1 mg of total RNA using random primers and the AMV reverse transcriptase (Promega). RT-qPCR was performed with the [Luna® Universal qPCR Master Mix](https://www.neb.com/products/m3003-luna-universal-qpcr-master-mix) (NEB) and a QuantStudio™ 5 Real-Time PCR System (Thermo Fisher Scientific). The relative amount of transcripts was normalized on GAPDH expression and determined with the DDCt method. The following primer sequences have been used:

| **GENE** | ***Forward*** | ***Reverse*** |
| --- | --- | --- |
| GAPDH | 5’-AATGGAAATCCCATCACCATCT-3’ | 5’-CGCCCCACTTGATTTTGG-3’ |
| IL-7R | 5'-GTGGCTATGCTCAAAATGGAGACT-3' | 5'-TGACATCTGGGTCCTCAAAAGC-3' |
| JAK2 | 5’-ATGGTCACCCACAGCAAGTTT-3’ | 5’- GCTGGTACTCGCTCTTGGAG-3’ |
| LAIR1 | 5'-GGACAACAGTCACAATGAGCAT-3' | 5'-TCTGATCTGGCGATGGAGG-3' |
| MCC | 5’GATGTCCAGGAGCGAACGA-3’ | 5’-CTTCCTCCCTGATGGTGGT-3’ |
| PIM1 | 5’-GGCAGAGGGTCTCTTCAGAAT-3’ | 5’-GGAGGTGGATCTCAGCAGTTT-3’ |
| PVT1 | 5'-TCATGGATTCTTACAGCTTGGATGTC-3' | 5'-GAAGAGTCGGGGTCTTACATTCC-3' |
| RAP2A | 5’-GCTTCATCCTCGTCTACAGCCT-3’ | 5’-CTGGCACTTTCTCATACCGCTTC-3’ |
| RNF157 | 5’- ACAGATGGAACTTTCTGTGTCAAGC-3’ | 5’-GTTATCACTCACTTCGTCTTCAGCC-3’ |
| SOCS2 | 5’-CGGTCAGACAGGATGGTACTG-3’ | 5’-AGTAGGTAGTTGAATGCGAGC-3’ |
| TNFRSF9 | 5'-AGCTGGTACATTCTGTGATAATAACAGGA-3' | 5'-TTGCTGGTGGAGGAACACTC-3' |
| VCAM1 | 5'-ATGTCAATGTTGCCCCCAGAG-3' | 5'-ACAGGATTTTCGGAGCAGGA-3' |
| ZBTB46 | 5'-CTCGCTGTCCCTGAATGAGTT-3' | 5'-CGCATGTGTCGCTTGAGGAT-3' |

**Supplementary Figure 1.** Flow cytometry analysis of a CD4+ T-LGLL patients. Leukemic cells are positive for CD3 and CD4 and express the CD8 marker at low intensity. The CD3+CD4+CD8dim population is also positive for the expression of CD57. This makes cytotoxic CD3+CD8+CD57+ T-cells from healthy controls (CTR) the closest cells to be considered as the normal counterpart of the leukemic population.


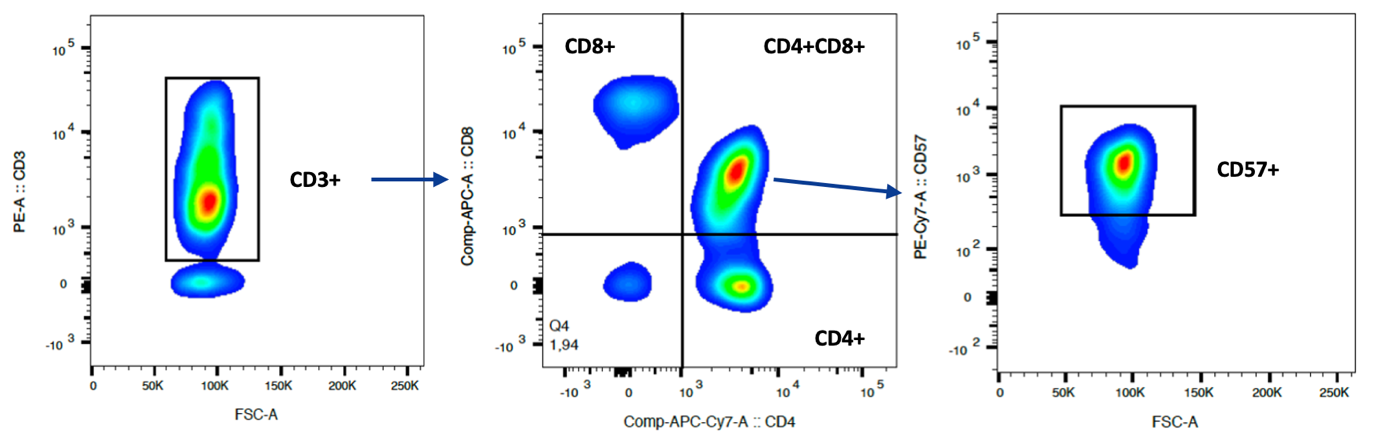


**Supplementary Figure 2.** Barplot of the LFC of the expression variation compared to controls of the genes dysregulated both in STAT3 mutated and in the other (OTH) T-LGLL cases. The line plot of the difference between the LFC values in the two groups indicates that the expression variation is more dramatic in the STAT3 mutated patients, in most cases.

**
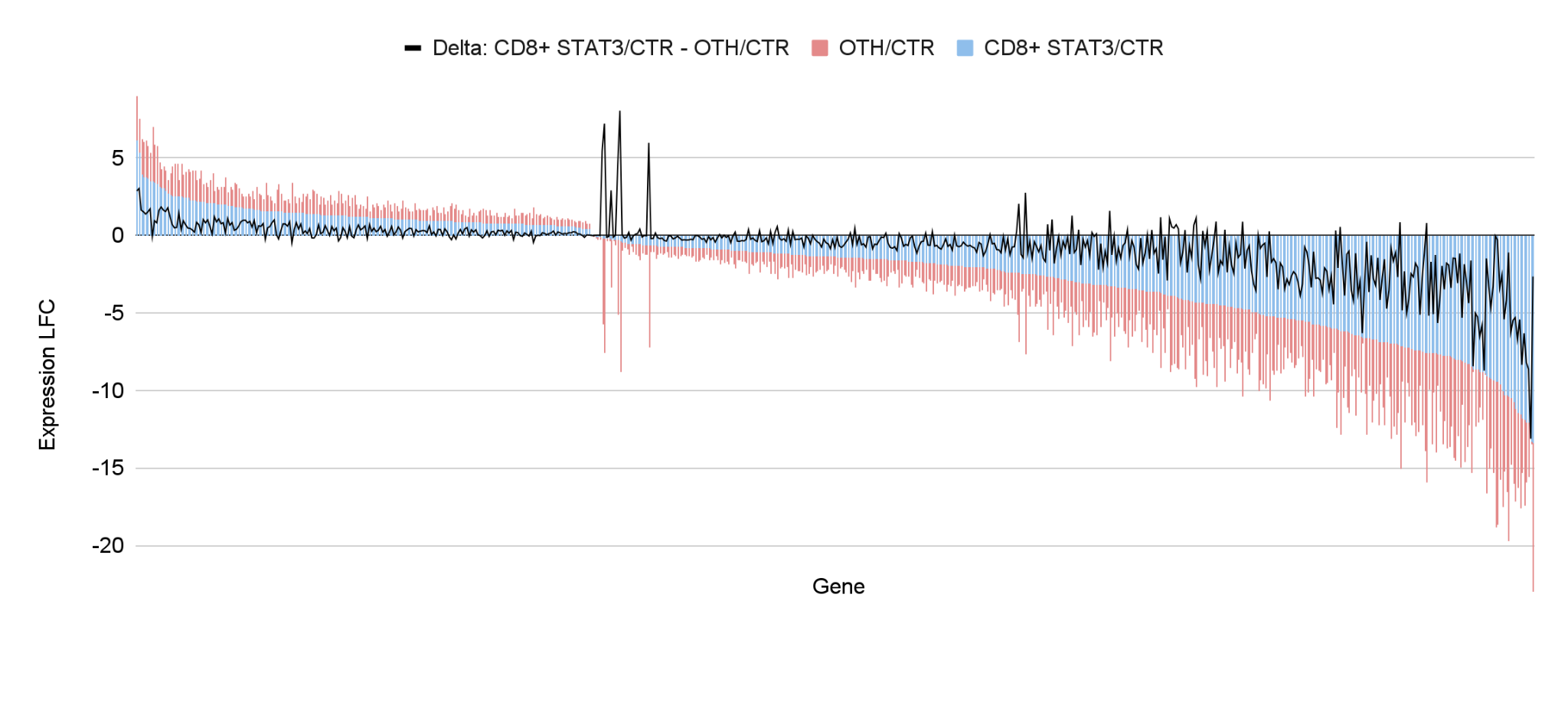
**

REFERENCES

1. [Van der Auwera GA, O’Connor BD.](http://paperpile.com/b/aLk9ty/Izfs) *[Genomics in the Cloud: Using Docker, GATK, and WDL in Terra](http://paperpile.com/b/aLk9ty/Izfs)*[. O’Reilly Media, 2020.](http://paperpile.com/b/aLk9ty/Izfs)
2. [Hagiwara K, Ding L, Edmonson MN, Rice SV, Newman S, Easton J](http://paperpile.com/b/aLk9ty/4kvU) *[et al.](http://paperpile.com/b/aLk9ty/4kvU)* [RNAIndel: discovering somatic coding indels from tumor RNA-Seq data. *Bioinformatics* 2020; 36: 4231.](http://paperpile.com/b/aLk9ty/4kvU)
3. [Coppe A, Andersson EI, Binatti A, Gasparini VR, Bortoluzzi S, Clemente M *et al.* Genomic landscape characterization of large granular lymphocyte leukemia with a systems genetics approach. *Leukemia* 2017; 31: 1243–1246.](http://paperpile.com/b/aLk9ty/YZ2n)
4. [Cheon H, Xing JC, Moosic KB, Ung J, Chan VW, Chung DS *et al.* Genomic landscape of TCRαβ and TCRγδ T-large granular lymphocyte leukemia. *Blood* 2022; 139: 3058–3072.](http://paperpile.com/b/aLk9ty/WnpJ)
5. [Marchand T, Lamy T, Loughran TP Jr. A modern view of LGL leukemia.](http://paperpile.com/b/aLk9ty/6RDm) *[Blood](http://paperpile.com/b/aLk9ty/6RDm)* [2024; 144: 1910–1923.](http://paperpile.com/b/aLk9ty/6RDm)
6. [Marchand T, Pastoret C, Moignet A, Roussel M, Lamy T. Large granular lymphocyte leukemia: a clonal disorder with autoimmune manifestations.](http://paperpile.com/b/aLk9ty/j6GG) *[Hematology Am Soc Hematol Educ Program](http://paperpile.com/b/aLk9ty/j6GG)* [2024; 2024: 143–149.](http://paperpile.com/b/aLk9ty/j6GG)
